# Statistical Analysis of Single-Trial Granger Causality Spectra

**DOI:** 10.1155/2012/697610

**Published:** 2012-05-10

**Authors:** Andrea Brovelli

**Affiliations:** Institut de Neurosciences de la Timone (INT), UMR 7289 CNRS, Aix Marseille University, Campus de Santé Timone, 27 Bd. Jean Moulin, 13385 Marseille, France

## Abstract

Granger causality analysis is becoming central for the analysis of interactions between neural populations and oscillatory networks. However, it is currently unclear whether single-trial estimates of Granger causality spectra can be used reliably to assess directional influence. We addressed this issue by combining single-trial Granger causality spectra with statistical inference based on general linear models. The approach was assessed on synthetic and neurophysiological data. Synthetic bivariate data was generated using two autoregressive processes with unidirectional coupling. We simulated two hypothetical experimental conditions: the first mimicked a constant and unidirectional coupling, whereas the second modelled a linear increase in coupling across trials. The statistical analysis of single-trial Granger causality spectra, based on *t*-tests and linear regression, successfully recovered the underlying pattern of directional influence. In addition, we characterised the minimum number of trials and coupling strengths required for significant detection of directionality. Finally, we demonstrated the relevance for neurophysiology by analysing two local field potentials (LFPs) simultaneously recorded from the prefrontal and premotor cortices of a macaque monkey performing a conditional visuomotor task. Our results suggest that the combination of single-trial Granger causality spectra and statistical inference provides a valuable tool for the analysis of large-scale cortical networks and brain connectivity.

## 1. Introduction

The study of linear dependence between time series is central in many fields of science inferring causal relations among components of complex systems. The notion of causality between two time series was introduced by Wiener [1] and was later formalised by Granger within the framework of multivariate autoregressive (MVAR) linear models [2]. The definition of Granger-Wiener causality is based on statistical prediction: a time series has causal influence on another if the variance of the autoregressive prediction error of the later is reduced by including the past measurements of the former. Geweke demonstrated that pairwise time-domain Granger causality can be additively decomposed by frequencies [3] and introduced measures of directional linear dependence between two time series conditioned on a third [4]. Recently, Dhamala et al. [5] showed that Granger causality spectra can be estimated from Fourier and wavelet transforms of time series data, in addition to parametric spectral estimation methods.

 In neuroscience, pairwise and conditional Granger causality spectra, based on parametric and nonparametric spectral methods, are becoming central for the analysis of interactions between neural populations within oscillatory brain networks [6–9]. In fact, current literature suggests that oscillations in neural populations activity, such as the local field potentials (LFPs), play a key role in modulating, filtering, and redirecting information in the nervous system [10–12]. Within this framework, Granger causality analysis is employed to reveal directional influences within oscillatory networks, such as during motor maintenance behaviours [13], and, more generally, it represents a crucial tool for the investigation of the neurophysiological substrate of cognitive functions [14].

 Conventional research in neuroscience employs single-trial-experimental designs and performs statistical inference on single-trial dependent variables. The study of large-scale neural interactions and oscillatory activity, as measured with electroencephalography or magnetoencephalography (EEG/MEG) data and local field potentials (LFPs), is no exception. In fact, neural correlates of cognitive functions are classically searched in modulations of signal power and phase synchrony between channels. Current tools allow the estimation of spectral measures using Fourier and wavelet transforms on a single trial basis [15, 16]. Statistical inference can then be performed within the framework of general linear models (GLM) [17]. Non-parametric (or distribution-free) inferential statistical methods are also used when no assumption about the probability distributions of the dependent variables can be made. However, it is currently unclear whether single-trial estimates of Granger causality spectra, as can be computed using non-parametric methods [4, 5], can be used reliably to assess directional influences among neural oscillations. In the current paper, we addressed this issue by combining single-trial Granger causality spectra with statistical inference based on the GLM approach. We assessed the suitability of the approach through the analysis of synthetic data consisting of a two-node network model with two autoregressive processes. In addition, we tested the tools on one exemplar neurophysiological recording session consisting of a pair of LFPs recorded simultaneously from the dorsal premotor and lateral prefrontal cortex of a macaque monkey performing a conditional visuomotor task. Overall, the results suggest that the combination of single-trial Granger causality spectra and statistical inference provides a useful tool for the investigation of brain connectivity. 

## 2. Materials and Methods

### 2.1. Synthetic and Neurophysiological Data

 To investigate whether directional coupling between bivariate signals can be inferred by combing single-trial Granger causality measures with parametric statistical tests, we analysed synthetic and neurophysiological data. Here is a description of the models used to generate synthetic data and the experimental methods of the neurophysiological recordings.

#### 2.1.1. Synthetic Data

**Figure 1:**
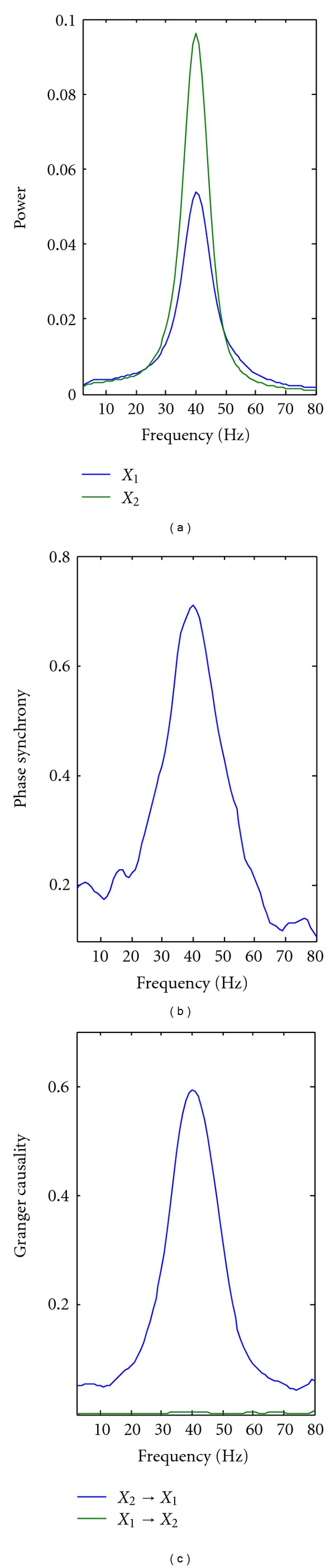
Simulations. Spectral analysis of synthetic data generated using a two-node network model with two autoregressive processes *X*_1_ and *X*_2_ and unidirectional coupling from *X*_2_ to *X*_1_. (a) shows the power spectra for *X*_1_ and *X*_2_, (b) shows their phase synchrony spectrum, and (c) depicts the Granger causality spectra.

 We considered a two-node network model with two autoregressive processes *X*_1_ and *X*_2_ and unidirectional coupling from *X*_2_ to *X*_1_:

(1) $$\begin{array}{c} X_{1}\left(t\right)=0.35\;X_{1}\left(t-1\right)-0.5\;X_{1}\left(t-2\right)+CX_{2}\left(t-1\right)+\in_{t},\\ X_{2}\left(t\right)=0.55\;X_{2}\left(t-1\right)-0.8\;X_{2}\left(t-2\right)+\xi_{t}, \end{array}$$

where  ∈_*t*_ and *ξ*_*t*_ are Gaussian white noise processes with zero means and unit variances, *C* is the coupling strength. The sampling frequency was considered to be 200 Hz (similar to [5]). The signals display a peak at 40 Hz in power and phase synchrony spectra (Figures 1(a) and 1(b), the left and central panel, resp.). From the construction of the model, we can see that *X*_2_ has a causal influence on *X*_1_ (Equation (1) and Figure 1(c)). We performed three sets of simulations. In all simulations, each trial comprised 100 points (500 msec of simulated data for a sampling rate of 200 Hz). In the first set of simulations, we mimicked a hypothetical experimental condition with a constant and unidirectional (i.e., from *X*_2_ to *X*_1_) coupling strength *C* = 0.3. We generated a data set containing 100 sessions, each composed of 50 trials. This data set was then used to characterise the sensitivity of the statistical analysis with respect to trial number in each session. In other words, we studied the statistical power of the tests by analysing data sets containing sessions with fewer trials (from 2 to 50 trials). In the second set of simulations, we generated synthetic data to investigate the range of coupling strengths that can be detected using the current approach. We generated 30 data sets (each containing 100 sessions and 50 trials) using coupling strengths *C* ranging from 0.01 to 0.3, in steps of 0.01 (30 possible values). In a third set of simulations, we modelled a linear increase in coupling across trials, as could be expected in experimental tasks exploring dynamic behaviours, such as during learning. We generated 100 sessions, each containing 150 trials, using coupling strength varying linearly across trials from 0 to 0.3 in steps of 0.3/*m,* where *m* was the number of trials in each session. For example, in the simulations with 150 trials, the coupling strength *C* at trial 1 was equal to 0, it increased linearly at a rate of 0.002 every trial (i.e., 0.3/150), and it was 0.3 at trial number 150. This dataset was also used to investigate statistical sensitivity as the number of trials in each session decreased from 150 to 4.

#### 2.1.2. Neurophysiological Data

 Neurophysiological recordings were conducted on a rhesus monkey at the Institut de Neurosciences Cognitives de la Méditerranée in the laboratory of Driss Boussaoud. Animal care and surgical procedures were in accordance with the European Communities Council Directive (86/609) for the use and care of laboratory animals in research. Results from literature suggest that the lateral prefrontal and dorsal premotor cortices play a key role in the acquisition and execution of arbitrary visuomotor associations (e.g., [18–20]). The aim of the electrophysiological study was to understand how these cortical areas coordinate during the acquisition and execution of arbitrary visuomotor associations. The entire neurophysiological database contains 93 recording sessions. In each session, the spiking activity of single neurons and the local field potentials (LFPs) were recorded from up to 4 tungsten micro-electrodes simultaneously placed in the lateral prefrontal (ventrolateral and dorsolateral prefrontal cortex, vlPFC and dlPFC, resp.) and dorsal premotor cortex (PMd). The analysis of the full dataset is beyond the scope of the current paper. However, the dataset represents an optimal benchmark to test our method on realistic neurophysiological bivariate data. Therefore, we analysed a pair of LFPs from one exemplar neurophysiological session.

**Figure 2:**
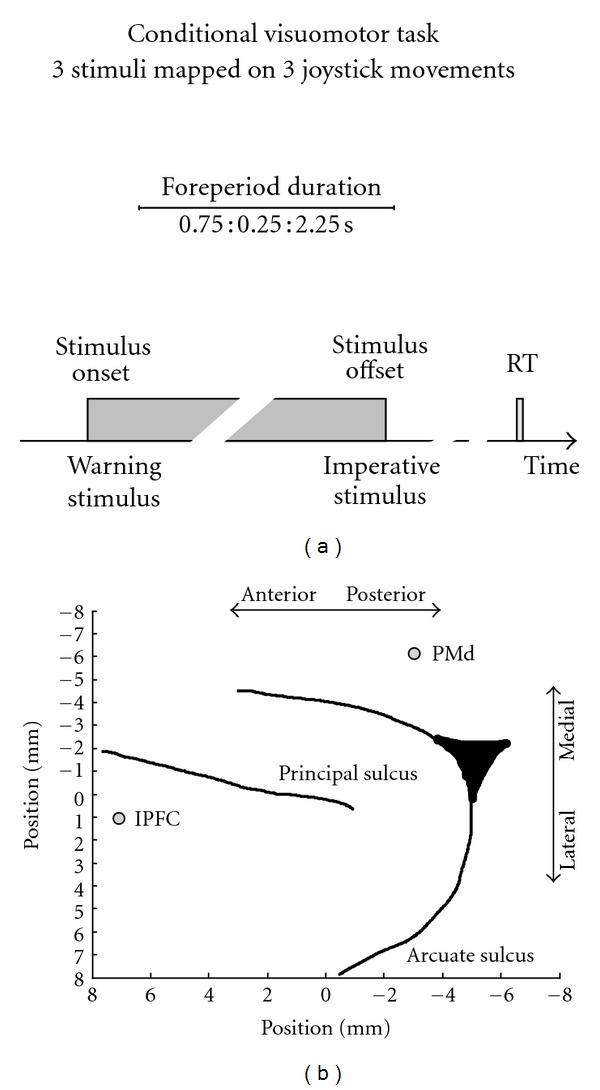
Neurophysiological experiments. (a) Conditional visuomotor task design. At each trial, the stimulus was presented at the centre of the screen (i.e., the warning stimulus) for a delay ranging from 0.75 to 2.25 seconds, in steps of 0.25 seconds (i.e., variable foreperiod paradigm). After stimulus offset (the instructive stimulus), the monkey had to execute the associated joystick movement to obtain reward. (b) Location of the two microelectrodes used to record the LFP analysed in the current paper. The electrodes were placed in the lateral prefrontal (lPFC) and dorsal premotor cortices (PMd).

 The behavioural task required the monkey to perform a conditional visuomotor task associating three abstract images to three joystick movements (Figure 2(a)). The task design conforms a variable foreperiod (FP) paradigm. Stimulus onset can be defined as the warning stimulus (WS), and its stimulus disappearance represents the imperative stimulus (IS) instructing the monkey to perform the action. The foreperiod duration (FP) is the time interval between the warning and imperative stimuli. A trial started when the animal held a joystick at a central position for 0.25 seconds. Thereafter, the stimulus was presented at the centre of the screen for a delay ranging from 0.75 to 2.25 seconds (the foreperiod duration), in steps of 0.25 seconds (7 possible delays). Delay durations were randomised across trials, and their offset instructed the monkey to execute the associated joystick movement, either to the right, up, or left. If movement direction was correct, a reward (fruit juice) was delivered after a fixed delay of 0.8 seconds; if incorrect, a purple circle appeared for 1.5 seconds as an error signal. The animal had 1 second to move the joystick in one of the three possible directions. If the response occurred late, the trial was aborted.

 Local field potentials (LFPs) were simultaneously recorded from two electrodes (the sampling rate was 1000 Hz, and the raw signals were band-pass filtered from 1 to 250 Hz) placed in the lateral prefrontal cortex (lPFC) and dorsal premotor area (PMd), respectively (Figure 2(b)). We analysed 259 epochs of 0.5 seconds in duration preceding the go cue (the imperative stimulus, IS) to present a single-case analysis illustrating how the neural oscillatory correlates of motor planning and/or expectation processes can be searched in the brain. To do so, we searched for trial-by-trial linear correlations between LFP spectral measures, such as power, phase synchrony, and Granger causality, and the IS expectancy. Imperative stimulus expectancy was estimated at each trial from the cumulative probability of IS occurrence, *P*_IS_. Since the foreperiod delays ranged from 0.75 to 2.25 seconds (in steps of 0.25 seconds), the cumulative probability of IS occurrence (*P*_IS_) was 1/7 for the shortest delay (0.75 seconds) and 1 for the longest (2.25 seconds). We then defined *S*_fp_ = −log(*P*_IS_) as the surprisal, or self-information, measuring the information content associated with IS occurrence. The surprisal in foreperiod duration *S*_fp_ is zero when the probability of occurrence of the go-signal *P*_IS_ is 1, that is, when the foreperiod duration is 2.25 seconds. In the analysis of the LFP, we correlated signal power, phase synchrony, and Granger causality at different frequencies using linear regression with the surprisal measure *S*_fp_ (more details in Section 2.3.3). 

### 2.2. Single-Trial Granger Causality Spectra

 To perform statistical inference based on parametric tests, we estimated Granger causality spectra on a single-trial basis. To do so, we computed the spectral density matrix for each trial using discrete fast Fourier transform (FFT) and Hanning window tapering of both synthetic and LFP time series data. The length of the FFT was 250 msec, stepped every 5 msec and zero-padded to 1 s to produce a frequency resolution of 1 Hz. Since each trial lasted 500 msec, the analysis produced 50 discrete FFTs for each trial. The spectra density matrix *S*(*n*)_*lm*_ at trial *n* at channels *l* and* m *(*l, m *= 1, 2 in our case) was then given by

(2) $$S{\left(n\right)}_{lm}={\langle X{\left(\tau ,n\right)}_{l,n}\cdot X{\left(\tau ,n\right)}_{m,n}^{*}\rangle }_{\tau },$$

where  *X*(*τ*,*n*)_*l*,*n*_ is the FFT of a 250 msec epoch of signal centred at time lag **τ** within trial *n*, the expectation (denoted by 〈⋯〉_*τ*_) is taken over all FFTs at different time lags **τ** within a trial and * denotes the complex conjugate. In other words, the “proper” ensemble averaging required to estimate the spectral matrix (e.g., equation 17.15 in [6]) is not performed over trials, but over time epochs within each trial. Note that we dropped the frequency suffix from the spectral measures for simplicity.

The single-trial spectra matrix *S*(*n*)_*lm*_ was then factorised using Wilson's algorithm to obtain the transfer function *H* and noise covariance matrix Σ [5, 8, 21, 22]. This step is critical in the estimation of Granger causality using non-parametric spectral analysis methods. The pairwise single-trial Granger causality spectra are then given by

(3) $$I{\left(n\right)}_{l\to m}=\frac{S{\left(n\right)}_{ll}}{S{\left(n\right)}_{ll}-\left(\Sigma_{mm}-\Sigma_{lm}^{2}/\Sigma_{ll}\right){\left|H{\left(n\right)}_{lm}\right|}^{2}}.$$

### 2.3. Statistical Analysis of Single-Trial Granger Causality Spectra

#### 2.3.1. General Linear Model Approach to Single-Trial Granger Causality Spectra

**Figure 3:**
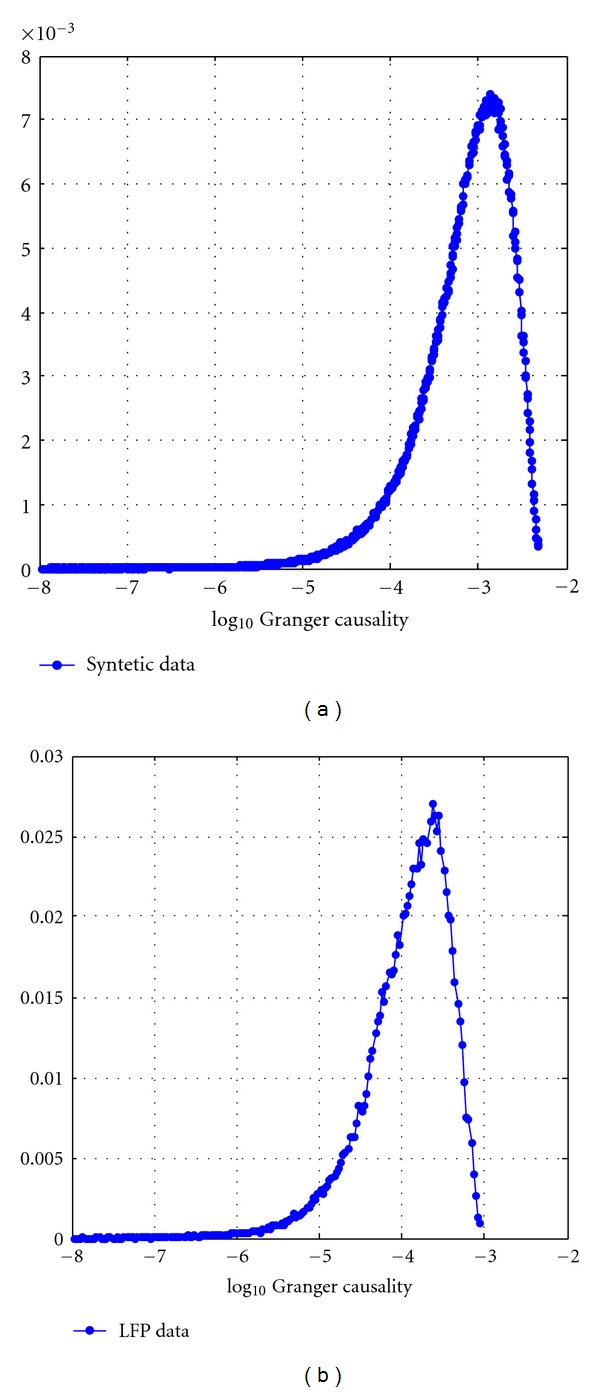
Distribution of log-transformed Granger causality data. Distribution of the log-transformation Granger causality values for synthetic (a) and LFP data (b). Histograms were computed using a number of bins equal to the square root of the number of elements in data.

 We adopted a general linear model (GLM) approach to analyse the single-trial Granger causality spectra. Granger causality measures issued from the synthetic and neurophysiological data are not normally distributed. Non-parametric statistical tests should be preferred. However, since that the GLM approach plays a key role in classical inference in neuroimaging and neurophysiology, we log-transformed Granger causality spectra to render the data approximately Gaussian (Figure 3) (refer also to [17]). The general linear model is normally expressed in matrix formulation,

(4) Y=Xβ+e,

where  *Y* is the dependent variable and is a column vector containing the data observations; *e* is a column vector of error terms; *β* is the column vector of model parameters (*β* = [*β*_1_,…,*β*_*p*_]^*T*^, where *p* is the number of model parameters); *X* is *j* × *p* design matrix, whose column vectors represent the independent variables. Model parameters  *β* were estimated using an ordinary least square method. In the current study, hypothesis testing and statistical inference were then performed using “contrast” vectors.

#### 2.3.2. Analysis of Synthetic Data

 We performed statistical analysis of single-trial Granger causality spectra computed from three simulations. The first data set was generated to simulate a constant and unidirectional coupling from *X*_2_ to *X*_1_. Our goal was to assess whether the underlying pattern of directional influence could be recovered from the statistical analysis of the data, in particular through the use of paired two-sample *t*-tests. The *t*-test assessed whether the mean values of log-transformed Granger causality spectra from *X*_2_ to *X*_1_ at a given frequency were significantly greater than from *X*_1_ to *X*_2_. Given that the synthetic data is generated using a unidirectional coupling from *X*_2_ to *X*_1_, there is justification for testing for significant difference specifically in one direction only (one-sided *t*-test). According to the nomenclature used above, we let *Y*(*j*) = [log⁡_10_⁡*I*(*n*)_2→1_,log⁡_10_⁡*I*(*n*)_1→2_]^*T*^ be a 2*n* × 1 column vector containing the two concatenated log-transformed single-trial Granger causality values at a given frequency and session (for simplicity, the suffices for frequency and session were dropped), where *j* = 1,…, 2*n* and *n* is the number of trials. The two-sample *t*-test is built using a design matrix *X* with two columns and *j* rows with variables indicating group membership (ones and zeros). We tested the hull hypothesis *H*_0_∶log⁡_10_⁡*I*(*n*)_2→1_ = log⁡_10_⁡*I*(*n*)_1→2_ against the alternative hypothesis *H*_1_∶log⁡_10_⁡*I*(*n*)_2→1_ > log⁡_10_⁡*I*(*n*)_1→2_ using the contrast *c* = [1−1]^*T*^. The *t*-test was performed at each frequency from 2 to 80 Hz and for each session. This procedure leads to 100 *t*-values and associated *P*-values at each frequency. To characterise the sensitivity of the statistical analysis with respect to the number of trials analysed in each simulated session, we performed *t*-tests on the log-transformed Granger causality values at 40 Hz (i.e., the peak frequency) and reduced the number of trials used in the statistical analysis, from 50 to 2 (i.e., 49 values). This produced 49 sets of 100 *t*- and *P*-values. Finally, we analysed the 49 sets of *P*-values to quantify the minimum number of trials required to detect unidirectional coupling from synthetic data. Assuming that the null hypothesis *H*_0_∶log⁡_10_⁡*I*(*n*)_2→1_ = log⁡_10_⁡*I*(*n*)_1→2_ is false (i.e., the synthetic data was generated using a unidirectional coupling strength *C* = 0.3 from *X*_2_ to *X*_1_); we estimated the probability to perform type II errors for each trial number (from 2 to 50 trials) at three significance levels **α** = 0.01, 0.001 and 0.0001. As a reminder, type II errors occur when a false null hypothesis is accepted. The probability of committing this kind of errors is defined as **β*,* and the power of the statistical test is 1−**β**. By convention, we used a minimum required power of 0.8 as a cutoff for the determination of the minimum number of trials required to obtain a significant discrimination of the directional coupling in the data. In our analysis, type II error, occurred if the estimated *P*-value exceeded the significance level **α*. *The probability **β** to perform Type II errors was estimated as the number of sessions displaying type II errors divided by the total number of sessions (100). The associated power was then given by 1–**β*. *This procedure was repeated for all trial numbers (from 2 to 50) so to give three curves of statistical power associated to three significance levels.

 The second set of simulations was generated to assess the ability to detect smaller coupling strengths. We analysed 30 data sets (each containing 100 sessions and 50 trials) each generated using different coupling strengths C varying from 0.01 to 0.3. We performed *t*-tests of the log-transformed Granger causality values at 40 Hz to test the hull hypothesis *H*_0_∶log⁡_10_⁡*I*(*n*)_2→1_ = log⁡_10_⁡*I*(*n*)_1→2_ against the alternative hypothesis *H*_1_∶log⁡_10_⁡*I*(*n*)_2→1_ > log⁡_10_⁡*I*(*n*)_1→2_ using the contrast  *c* = [1−1]^*T*^. The *t*-test was repeated at all coupling strengths values to produce 30 sets of 100 *t*-values and associated *P*-values. To estimate the minimum coupling strength *C* detectable with the current approach, we performed power analysis (described in the previous paragraph) as a function of coupling strength at three levels of significance **α** = 0.01, 0.001, and 0.0001.

 In the third set simulations, we modelled a linear increase in coupling strength *C* across trials. The simulations generated 100 sessions, each containing 150 trials. To retrieve the correct pattern of directional influence, we performed linear regression analysis. We let *Y*(*n*) = [log⁡_10_⁡*I*(*n*)_2→1_]^*T*^ be a *n* × 1 column vector containing the log-transformed single-trial Granger causality values at 40 Hz, where *n* is the number of trials. The design matrix *X* contained two columns and *n* rows. The first column modelled baseline and contained only ones, whereas the second column contained the values of the actual coupling strengths *C* as they varied across trials. The linear regression was then tested using a contrast *c* = [01]^*T*^. To assess the sensitivity of the statistical analysis with respect to the number of trials analysed in each session, we reduced the number of trials used in the statistical analysis, from 150 to 3 stepped every trial (i.e., 148 values). This produced 148 sets of 100 *t* and *P*-values. The minimum number of trials required to detect significant effects was estimated using the power analysis at three significance levels **α** = 0.01, 0.001, and 0.0001.

#### 2.3.3. Analysis of Neurophysiological Data

 We studied the relevance for neurophysiology by analysing two local field potentials (LFPs) simultaneously recorded from the prefrontal and premotor cortices of a macaque monkey performing a conditional visuomotor task. As mentioned in Section 2.1.2, the monkey performed a conditional visuomotor task based on a variable foreperiod (FP) paradigm. We searched for linear correlations between the surprisal in foreperiod duration *S*_fp_ (i.e., −log(*P*_IS_)) and modulations in signal power, phase synchrony, and Granger causality at each frequency. Spectral analysis of LFP data was performed using the same parameters used for synthetic data. Briefly, the LFP power, phase synchrony, and Granger causality spectra were computed at single trials in a time window of 500 msec preceding the imperative stimulus (IS), using FFT sliding windows of 250 msec, stepped every 5 msec and zero-padded to 1 s. We performed linear regression analysis at each frequency between the trial-by-trial modulations in LFP spectral estimates (i.e., power, phase synchrony, and Granger causality) and the surprisal in foreperiod duration *S*_fp_ using the GLM approach described above. In other words, we let *Y*(*n*) be an *n* × 1 column vector containing the spectral measures (the log-transformed power, phase synchrony, Granger causality values), where *n* is the number of trials. The design matrix *X* contained two columns and *n* rows. The first column modelled baseline and contained only ones whereas the second column contained the values of the surprisal in foreperiod duration *S*_fp_ as they varied across trials. Since *S*_fp_ scales negatively with respect to the expectancy of IS occurrence, we tested a negative linear regression using a contrast *c* = [0−1]^*T*^. At each frequency, we obtained a *t*-value and associated *P*-value.

#### 2.3.4. Software Implementation

 All simulations and analyses were performed on MATLAB software (The MathWorks, Inc.). Spectral analysis was performed using functions from EEGlab software (http://sccn.ucsd.edu/eeglab/), whereas spectral matrix decomposition was performed using functions implemented in FieldTrip software (http://fieldtrip.fcdonders.nl/). Statistical analyses were performed using Matlab Statistical Toolbox.

## 3. Results and Discussion

### 3.1. Synthetic Data

**Figure 4:**
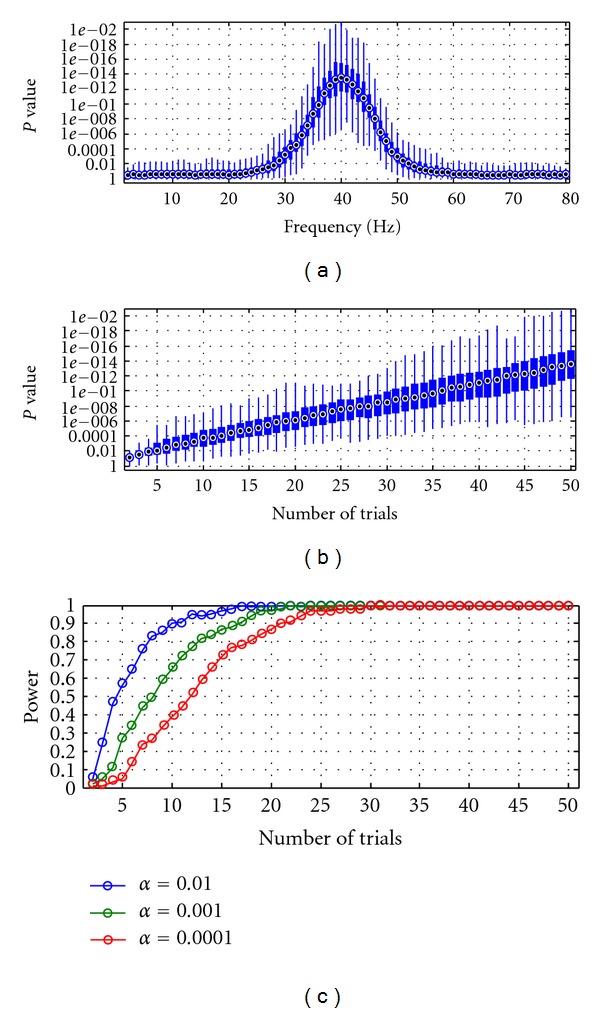
Simulation 1. (a) Granger causality *p*-spectra. Boxplot representation of the distribution of the *P-*values associated with each *t*-test. At each frequency, the circle in the box is the median value, the edges of the box are the 25th and 75th percentiles, and the whiskers extend to the most extreme data points. The *P*-values in each boxplot are associated with *H*_1_∶log⁡_10_⁡*I*(*n*)_2→1_ > log⁡_10_⁡*I*(*n*)_1→2_. (b) Boxplot of the *P-*values plotted over the number of trials simulated in each session. (c) Statistical power curves at three levels of significance **α** = 0.01, 0.001 and 0.0001. By convention, the minimum required power of 0.8 was set as a cutoff for the determination of the minimum number of trials required to obtain a significant discrimination of the directional coupling in the data.

 We simulated a two-node network model with two autoregressive processes *X*_1_ and *X*_2_, and unidirectional coupling from *X*_2_ to *X*_1_ (1). The sampling frequency was considered to be 200 Hz, leading to signals with a peak at 40 Hz in the power and phase synchrony spectra (Figure 1(a) and 1(b), resp.). From the construction of the model, *X*_2_ had a linear causal influence on *X*_1_ (Equation (1) and Figure 1(c)). To investigate whether directional influence between bivariate signals could be inferred by combining single-trial Granger causality measures with statistical inference methods, we performed three simulations. In the first set of simulations, we mimicked a hypothetical experimental condition with a constant coupling across trials (i.e., *C *= 0.3). We then performed paired two-sample *t*-tests between the log-transformed Granger causality spectra to assess whether the mean values of log-transformed Granger causality spectra from *X*_2_ to *X*_1_ at a given frequency were significantly greater than from *X*_1_ to *X*_2_. The *t*-test was performed at each frequency from 2 to 80 Hz and for each session (100 in total). This leads to 100 *t*-values and associated *P*-values at each frequency (see Section 2.3.2).  Figure 4(a) depicts the boxplot representation of the distribution of the 100 *P-*values at each frequency. The results show that the statistical analysis is able to correctly infer the directional influence from *X*_2_ to *X*_1_. In fact, the boxplots peak at 40 Hz and the *P-*values at 40 Hz are less than 10^−6^ (i.e., highly significant). To characterise the sensitivity of the statistical analysis with respect to the number of trials analysed in each session, we performed *t* tests on the log-transformed Granger causality values at 40 Hz (i.e., the peak frequency) and reduced the number of trials used in the statistical analysis, from 50 to 2.  Figure 4(b) shows the boxplot of the *P-*values over the number of trials simulated in each session. As expected, the mean *P*-values increase as the number of trials is reduced. To determine the minimum number of trials required to detect unidirectional coupling from synthetic data, we performed statistical power analysis (see Section 2.3.2). Briefly, statistical power is defined as 1−**β**, where **β** is the probability to perform type II errors (i.e., acceptance of a false null hypothesis) for three significance levels **α** = 0.01, 0.001, and 0.0001. The minimum number of trials required is defined as the first trial number whose statistical power exceeds 0.8.  Figure 4(c) shows the statistical power for three significance levels. The minimum number of trials required for a statistical significance of 0.01, 0.001, and 0.0001 was 8, 13 and 18, respectively. To conclude, the results from the first simulation suggest that the combination of statistical inference based on parametric tests, such as *t* test, with single-trial Granger causality spectra successfully recovers the underlying pattern of directional influence with a limited number of trials.

**Figure 5:**
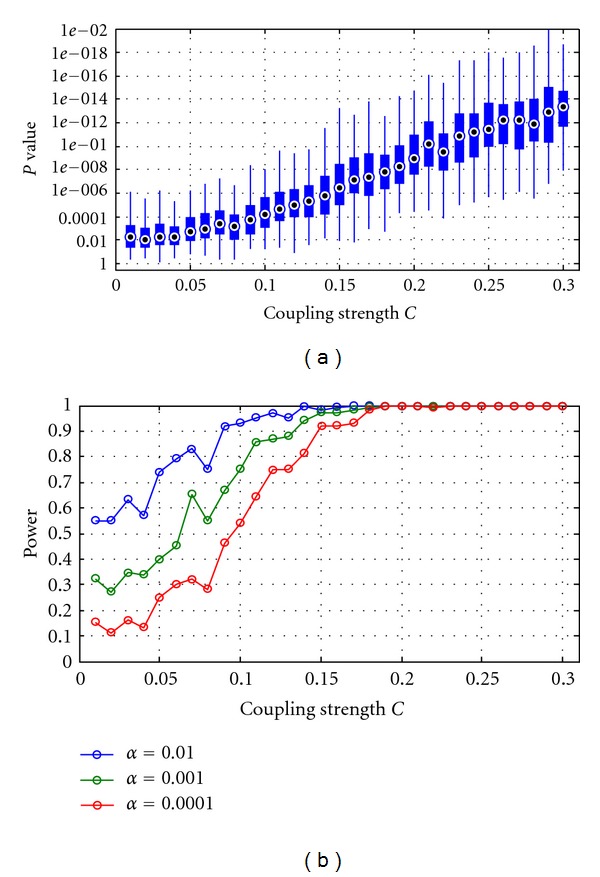
Simulation 2. (a) Granger causality *p*-spectra. Boxplot representation of the distribution of the *P-*values associated with each *t*-test as a function of the coupling strength *C*. (b) Statistical power curves at three levels of significance **α** = 0.01, 0.001, and 0.0001 over the coupling strength *C*.

 In the second simulations, we investigated the range of coupling strengths that can be detected using the current approach. We generated synthetic data using coupling strengths varying from 0.01 to 0.3.  Figure 5(a) shows the boxplot of the *P*-values as a function of coupling strength *C*. The *P*-values increase as the coupling strengths are decreased. To estimate the minimum coupling strength *C* detectable from synthetic data, we performed statistical power analysis (as described in the methods section) for three levels of significance **α** = 0.01, 0.001, and 0.0001.  Figure 5(b) shows the statistical power curves, and it shows that the minimum coupling strengths detectable at statistical significances of 0.01, 0.001, and 0.0001 were 0.09, 0.11, and 0.14, respectively. The results therefore pointed out the limitation of the current approach to relatively high values of coupling strengths for a limited number of trials (i.e., 50 as in this case). However, for coupling strength greater than 0.14, the results suggest that directionality can be estimated with high significance.

**Figure 6:**
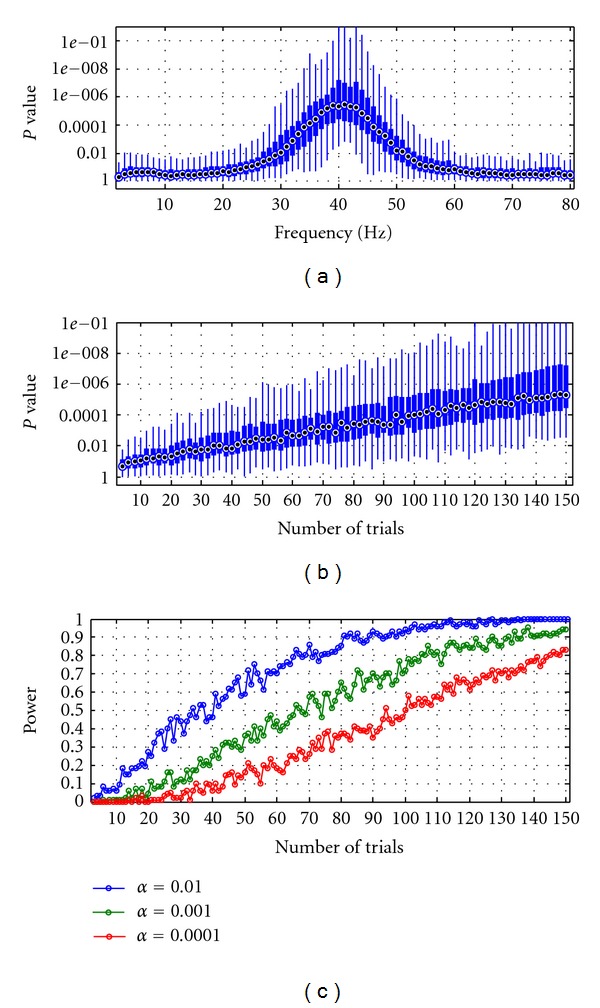
Simulation 3. (a) Granger causality *p*-spectra. Boxplot of the *P-*values associated with the linear regressions performed at each frequency between the log-transformed Granger causality spectra and the coupling strengths varying across trials. (b) Boxplot of the *P*-values plotted over the number of trials simulated in each dataset. (c) Statistical power curves at three levels of significance **α** = 0.01, 0.001, and 0.0001 as a function of the number of trials used in each session.

 In the third simulations, we modelled a linear increase in directional influence from *X*_2_ to *X*_1_ varying from 0 to 0.3 across 150 trials (as could be expected in dynamic experimental tasks, such as during learning tasks). We performed linear regressions on a trial-by-trial basis between the log-transformed Granger causality spectra from *X*_2_ to *X*_1_ and the coupling strengths.  Figure 6(a) shows the Granger *p*-spectra displaying the boxplot representation of the *P-*values associated with the linear regressions performed at each frequency. The boxplots show that the statistical analysis based on linear regression recovers the expected pattern of directional influence from *X*_2_ to *X*_1_. Statistical *P-*value less than 0.01 are observed at the peak frequency of 40 Hz. To better quantify the minimum number of trials in each session required to significantly detect directional influence from the data, we performed linear regressions on the log-transformed Granger causality measures at 40 Hz (i.e., the peak frequency) on datasets containing fewer and fewer trials (from 150 to 4 trials).  Figure 6(b) shows the boxplot of the *P-*values over the number of trials simulated in each dataset. Statistical power analysis showed that the minimum number of trials required for a statistical significance of 0.01, 0.001, and 0.0001 were approximately 70, 110 and 145, respectively (Figure 6(c)). Therefore, the results suggest that to detect linear increases in coupling strengths across trials using linear regression, the minimum number of trials is larger than the number of trials required to detect constant directionality (Figure 4(c)).

 The results of the simulations showed that single-trial estimates of Granger causality spectra can be used to quantify directional influence between bivariate synthetic data when combined with statistical inferences based on the GLM approach, such as *t*-tests (Figures 4(a) and 5(a)) and linear regression (Figure 6(a)). As expected, the method show sensitivity with respect to the number of trials used for statistical analysis (Figures 4(b) and 6(b)) and the coupling strength *C* (Figure 5(b)). The lower the number of trials and coupling strength, the less significant is the statistical analysis. The simulations quantified the minimum number of trials (Figure 4(c) and Figure 6(c)) and the range of coupling strengths (Figure 4(c)) required to detect directionality at different significance levels. To conclude, the simulation studies indicate that statistical inference based on general linear models (GLM) in combination with single-trial Granger causality spectra is a valuable tool to infer directional coupling among bivariate signals. Most importantly, the results suggest that the full range of statistical methods based on parametric (e.g., analysis of variance (ANOVA)) and non-parametric tests, general and generalized linear models, can be used to analyse single-trial Granger causality spectra issued from neurophysiological experiments. Even though the current work focused on Granger causality measures, we should stress that alternative methods can be used to quantify directional interactions, such as partial directed coherence (PDC), directed transfer function (DTF), directed transfer function (DTF), and transfer entropy (TE). While a detailed evaluation of the reliability of these measures was beyond the scope of the current paper (e.g., [23, 24]), we suggest that our simple statistical approach may be applied to alternative measures of directional coupling.

### 3.2. Neurophysiological Data

**Figure 7:**
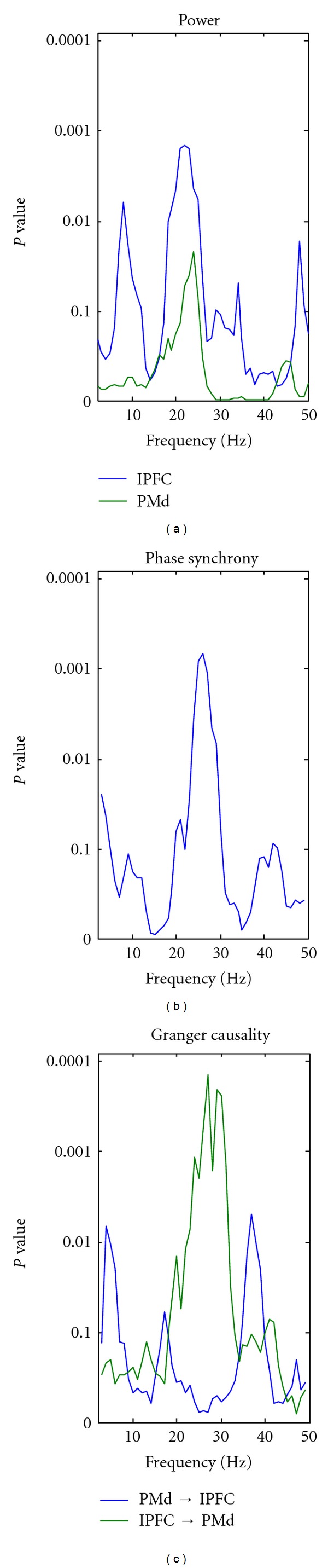
Neurophysiological results. Spectral analysis of neurophysiological data displaying *p*-spectra for (a) LFP power, (b) phase synchrony, and (c) Granger causality.

 To investigate the feasibility of the current approach on realistic data, we analysed an exemplar neurophysiological session. Given that the monkey performed a conditional visuomotor task based on a variable foreperiod (FP) paradigm, we searched for linear correlations between the surprisal in foreperiod duration *S*_fp_ (i.e., −log(*P*_IS_)) and modulations in signal power, phase synchrony, and Granger causality in a time window of 500 msec preceding the imperative stimulus (IS). At each frequency, we performed linear regression analysis between the trial-by-trial modulations in power, phase synchrony, and Granger causality with *S*_fp_. Given that *S*_fp_ scales negatively with the probability of occurrence of the go cue (i.e., IS expectancy), we searched for negative linear correlations.  Figure 7 shows the *p-*spectra associated with the linear regressions. Signal power in the lateral prefrontal cortex (blue curve Figure 7(a)) shows linear negative correlation with respect to *S*_fp_, mainly in the beta range (from 15 to 30 Hz). A similar tendency is observed for the LFP in the PMd cortex, although not significant (green curve in Figure 7(a)). Phase synchrony between the LFPs in the two cortical areas displays significant effects in the same frequency band, peaking around 25 Hz (Figure 7(b)). This indicates that the two areas oscillated synchronously in the beta range, and the degree of coherence scaled with the probability of occurrence of the go-cue. We then correlated the Granger causality measures with the values of *S*_fp_, and we found that the coherence between the LFPs recorded in the two cortical areas can be explained by a unidirectional Granger causality influence from the lateral prefrontal cortex to the dorsal premotor area. The amount of Granger causality scales negatively with *S*_fp_. This shows that the directional influence among the two areas increases with time as the probability of occurrence of the go cue increases. The prefrontal and premotor cortices, in addition to the basal ganglia, supplementary motor area, and cerebellum, have all been linked to the explicit estimation of duration [25]. Even though no conclusion can be drawn from the analysis of a single neurophysiological session, the current single-case analysis suggests a top-down effect of the lateral prefrontal cortex onto the dorsal premotor area. Further analyses of the full neurophysiological dataset are required to better understand the dynamic interplay between the prefrontal and premotor cortices in the prediction and update of temporal expectations as foreperiod unfolds. Overall, as an approach to large-scale cortical network analysis, our results suggest that statistical analyses of single-trial Granger causality spectra provides a valuable tool for in-depth investigation of the functional coupling of distributed neuronal assemblies.

## 4. Conclusions

 The analysis of the synthetic data showed that directional coupling between bivariate signals can be inferred by combing single-trial Granger causality measures with parametric statistical tests based on a GLM approach. The statistical analysis of single-trial Granger causality spectra, based on *t*-tests and linear regression, successfully recovered the underlying pattern of directional influence. In addition, we characterised the minimum number of trials and coupling strengths required for significant detection of directionality. In fact, the number of trials required to obtain significant corresponds to conventional experimental situations. Finally, we demonstrated the relevance for neurophysiology by analysing two local field potentials (LFPs) simultaneously recorded from the prefrontal and premotor cortices of a macaque monkey performing a conditional visuomotor task. Our results suggest that the combination of single-trial Granger causality spectra and statistical inference provides a valuable tool for the analysis of large-scale cortical networks and brain connectivity. We suggest that the current approach may represent a simple statistical tool useful for the analysis of neurophysiological recordings issued from electroencephalographic (EEG), magnetoencephalographic (MEG), and intracranial EEG experiments. The approach may be extended to the full range of statistical methods based on parametric (e.g., analysis of variance (ANOVA)) and non-parametric tests, general and generalized linear models. Finally, we suggest that the same approach may be applied to alternative measures of directional coupling based on the Granger causality principle.
